# Renal Cell Carcinoma with IVC Thrombi; Current Concepts and Future Perspectives

**DOI:** 10.4137/cmo.s464

**Published:** 2008-03-26

**Authors:** Mohammed Ahmed Abdel-Muneem Nouh, Masashi Inui, Yoshiyuki Kakehi

**Affiliations:** 1 Department of Urology, Kagawa University, Faculty of Medicine, Kagawa, Japan; 2 Department of Urology, Sohag University, Sohag faculty of Medicine, Sohag, Egypt

**Keywords:** RCC, tumor thrombi, angioscopy

## Abstract

The incidence of venous extension to the inferior vena cava (IVC) in renal cell carcinoma (RCC) is markedly increased recently mostly due to the advances in diagnostic modalities. Such vascular invasion implies a heightened biologic behavior and a surgical challenge during the course of treatment. In this study, we reviewed the classification guidelines, recent diagnostic tools and up-to-date therapeutic modalities for RCC with IVC tumor thrombi added to the prognostic significance regarding the pathologic nature of vascular invasion; cephalad extent of thrombi and any associated distant metastasis. Also, we are providing our suggestion regarding the use of angioscopy for removal of IVC thrombi in a relatively bloodless field without aggressive surgical manipulations or shunt techniques for maintenance of hemodynamic stability.

## Introduction

Venous migration and tumor thrombus formation are unique aspects of renal cell carcinoma (RCC)^1^ with significant therapeutic and prognostic implications.^2^ It has been reported to occur in 4%–10% of patients with renal neoplasms.^3^ Within this group, 2%–16% have tumors extending into the right atrium.^4^ The tumor thrombus may invade the caval wall which is difficult, if not impossible, to predict preoperatively.^1^ In this study; the classification, recent diagnostic tools and therapeutic modalities for the IVC thrombi in patients with RCC are reviewed. Also, controversial studies regarding the prognostic significance of such thrombi and their cephalad extent in the venous system will be discussed. Finally, a new technique regarding the use of angioscopy for removal of caval thrombi under direct vision is highlighted for future studying.

## Classification

**Figure 1:**
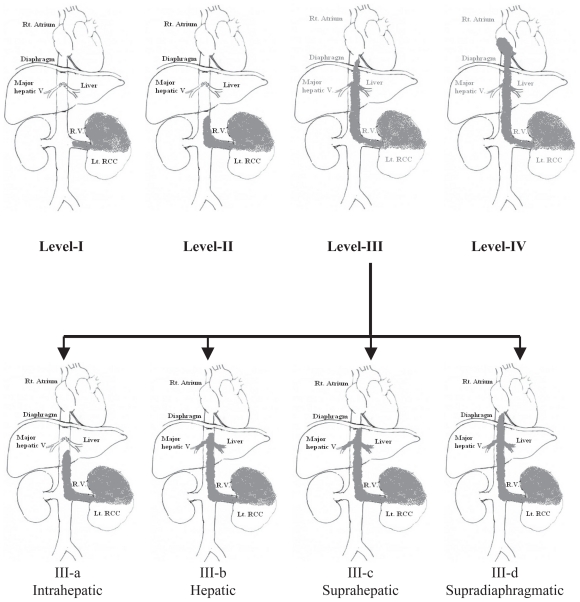
Classification of RCC with IVC thrombi showing the sub-divisions of group-III thrombi.

The traditional classification of RCC caval thrombi is as follows (Fig. 1)^3^:

Group I: Venous thrombus in the renal vein not reaching IVC.
Group II: Infra-hepatic IVC thrombus.
Group III: Thrombi in retrohepatic or suprahepatic IVC not reaching the right atrium. Because of the prognostic relevance of the cranial intracaval neoplastic extension and the surgical consequences related to IVC exposure as well as the degree of vascular control required to extract these thrombi,^5^ group III is further subdivided according to the anatomical relation of the thrombus to the major hepatic veins^6^:-
  IIIa (intrahepatic): A thrombus extending into the retrohepatic IVC but below the ostia of major hepatic veins.
  IIIb (hepatic): A thrombus extending into the retrohepatic IVC reaching the ostia of the major hepatic veins and may extend into them causing Budd-Chiari syndrome.
  IIIc (suprahepatic, infradiaphragmatic): A thrombus extending into the retrohepatic IVC above the major hepatic veins but below the diaphragm.
  IIId (suprahepatic, supradiaphragmatic and infra-atrial): A thrombus extending into the supradiaphragmatic, intrapericardial IVC but not into right Atrium.
Group IV: Right atrial thrombi.

## Diagnosis

The progressively rising incidence of RCC has, in part, been attributed to early detection of tumors by the widespread use of abdominal imaging modalities as ultrasound, computed tomography (CT) and magnetic resonance imaging (MRI).^7^ The overall goal of preoperative imaging is to determine the malignant potential of the lesion, assess tumor size and location, determine capsular invasion, and identify regional lymphadenopathy or metastases for accurate staging. In addition, the renal vein and IVC need to be assessed for tumor thrombi.^8^

**Figure 2:**
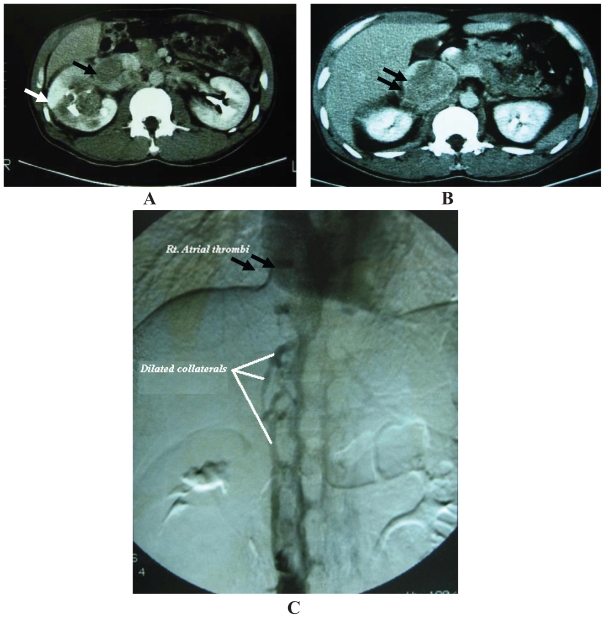
**A**: Abdominal CT showing Rt. RCC (white arrow) with a large tumor thrombus extending into the IVC (black arrow). **B**: Abdominal CT for the same patient’s Rt. kidney (Upper pole) with a large tumor thrombus extending more cephalad in the IVC (black arrow). **C**: Venacavography (for the same patient) showing dilated IVC collaterals due to a large tumor thrombus extending into the IVC (white arrows) up to right atrium (black arrows).

Traditionally, venacavography was the gold standard for detection of tumor thrombi (Fig. 2-C).^9^ However, it is rarely used recently as it is not able to demonstrate caval wall invasion by the neoplastic process and is not additive to ultrasonography, CT, and MRI added to its associated high risk of contrast-induced renal injury.^4^

Ultrasound is the first imaging modality used to evaluate patients with RCC. However, its sensitivity in detecting tumor thrombi depends on the position of the thrombus with a lower sensitivity for infrahepatic than for intrahepatic thrombi (68% and 100% respectively).^10^

CT is extremely useful in demonstrating the cephalad extent of the thrombus and occult metastasis in the majority of cases (Fig. 2-A&B).^4^ Venous extension is optimally visualized during the corticomedullary phase of enhancement, when contrast enhancement of the renal vein is at its peak, as a low density-filling defect within the vein. Enhancement pattern of thrombi helps to distinguish tumor thrombi from bland ones. However, conventional CT has a lower sensitivity in delineating the superior margin of the thrombus when compared to MRI (76% and 100% respectively). The evolution of multidetector (MS) CT; which provides a three-dimensional rotational CT image, improved CT sensitivity in RCC (up to 95%)^11^ by accurately demonstrating the relationship of the renal mass with the renal tissue and adjacent organs and vessels and facilitating detection of tumor thrombi and distant metastases hence accurate staging and operative planning.^12^ The drawbacks of MS CT include (1) renal vein tapering and failure of visualization of tumor thrombi due to failure of contrast medium inflow to the IVC in the presence of an extensive tumor thrombus or by an external renal vein or caval compression by an adjacent metastatic tumor or enlarged lymph nodes and (2) failure to precisely delineate the cephalad extent of the thrombus as blood and thrombus radiological densities can be similar.^10^

Currently, MRI is the gold standard for delineating the level and extent of tumor thrombi in the IVC and staging of RCC with a sensitivity of 96–100% and it may rule out caval wall invasion so the exact surgical procedure can be planned.^13^,^14^ It is proved to be superior to MS CT for thrombus detection, delineating its cephalad level and staging of RCC because of its ability to have a free imaging plane with an optimal spatial resolution in the sagittal and coronal planes.^14^ Also, it has intrinsic contrast superiority to CT and does not need to rely on contrast medium to differentiate tumor thrombus from blood. However, recent studies showed that MRI diagnosis of a tumor thrombus is not significantly better than that of MS CT.^13^

The diagnostic effect of Positron Emission Tomography (PET) is dependant on the abnormal uptake of 18-fluoro-2-deoxyglucose (FDG) by the tumor tissues and its hypermetabolic thrombi with a sensitivity of 77%–94% in RCC.^15^ The drawbacks of PET in RCC include: (1) FDG is renally excreted without significant tubular reabsorption. This mode of excretion results in collecting system accumulation and makes the identification of parenchymal lesions more difficult. Most primary renal tumors have moderate but variable uptake of 18F-FDG when compared with metastatic lesions.^16^ (2) its ability to demonstrate modest FDG uptake in granulation tissues and inflammatory venous processes including catheter-induced thrombi and septic thrombophlebitis that may be misinterpreted as malignancy.^15^,^17^ So, positive PET venous findings require correlation with the clinical history and supplemental cross-sectional imaging to determine the exact nature of the thrombus for adequate therapy. Recent studies reported the significance of using a combined PET-CT imaging system to gain the diagnostic benefits of both investigative techniques in anatomic localization and definition of RCC and the level and pathologic nature of any associated IVC thrombi which may be equivalent to or more sensitive than MRI.^17^

Duplex scanning and trans-esophageal echocardiography can be performed pre- and intraoperatively to confirm the cephalad extent of tumor thrombus and to evaluate cardiac function especially for level IIId and IV thrombi.^18^

## Surgical Principles

*Surgical objectives*: (1) Complete resection of tumor and its caval thrombi, (2) prevention of tumor embolism by temporary placement of an IVC filter or plication of the IVC in its suprahepatic or retrohepatic segment,^18^ (3) minimizing blood loss, (4) maintenance of hemodynamic stability, and (5) prevention of vital organ ischemia.

*Incision and retraction*: for infrahepatic and intrahepatic IVC thrombi, a midline transperitoneal incision provides a generous access to the IVC. Bilateral subcostal approaches have also been used for equivalent exposure.^8^ As the thrombus level migrates cephalad, a thoraco-abdominal approach is warranted which entails a triradiate incision including two subcostal incisions extending out lateral to the mid axillary line and a vertical midline incision up to the xiphoid process. A self-retaining retractor is placed to elevate the costal margins and splay them laterally toward the axillae which helps to flatten the diaphragm and enables easier liver mobilization and access to the retrohepatic space in cases with thrombi extending in the suprahepatic IVC. In group IIId and IV thrombi; the diaphragm is cut in the midline at the IVC hiatus then the pericardium is opened to visualize the intrapericardial IVC and the right atrium. The implementation of this minimal access versus a thoracic approach avoids the traditional midline sternotomy and has been paramount in reducing surgical morbidity.^8^

**Figure 3:**
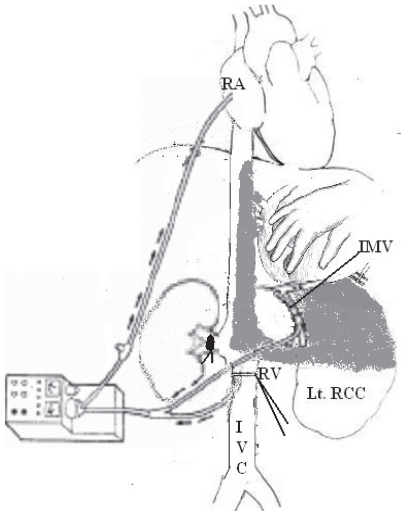
Diagrammatic illustration for the pump-driven veno-venous bypass (VVB) technique. **Abbreviations:** RA: Right Atrium; IMV: Inferior mesenteric vein; RV: Renal vein.

*Vascular control*: After removal of the affected kidney, careful ligation of IVC lumbar tributaries that tether it to the posterior abdominal wall is mandatory to achieve circumferential vascular control of IVC.^19^ Additionally; this maneuver allows liver rotation under IVC, thus, enabling exploration of the hepatic ostia end-on for any thrombus extension. However, it markedly reduces the venous return causing profound hypotension. Hemodynamic stability is restored by: (1) creating a veno-venous bypass in which IVC distal to the thrombus is cannulated with inflow at the level of the right atrium or cephalic vein.^20^ With a more cephalad extent of tumor thrombi, a pump-driven veno-venous bypass (VVB) can be done in which the IVC and the inferior mesenteric vein (for portal decompression) are connected to the right atrium (Fig.3).^21^ This technique allows a prolonged caval cross-clamping without affecting the hemodynamic status and avoids the surgical consequences of cardiopulmonary bypass (CPB) techniques. (2) Concomitant partial or total cross-clamping of the abdominal aorta. This technique will maintain systemic blood pressure above 100 mmHg.^18^ The infrarenal or the supracoeliac segments of abdominal aorta can be clamped. However, the later should not be continued for more than 30 minutes to avoid renal dysfunction of an only functioning kidney. (3) CPB with or without deep hypothermic circulatory arrest (DHCA) is used to maintain hemodynamic stability especially for level IIId and IV tumor thrombi.

*Extraction of tumor thrombi*: Further surgical approach is dependant on the cephalad extent of the tumor thrombus within the IVC.

### Group I and II

**Figure 4:**
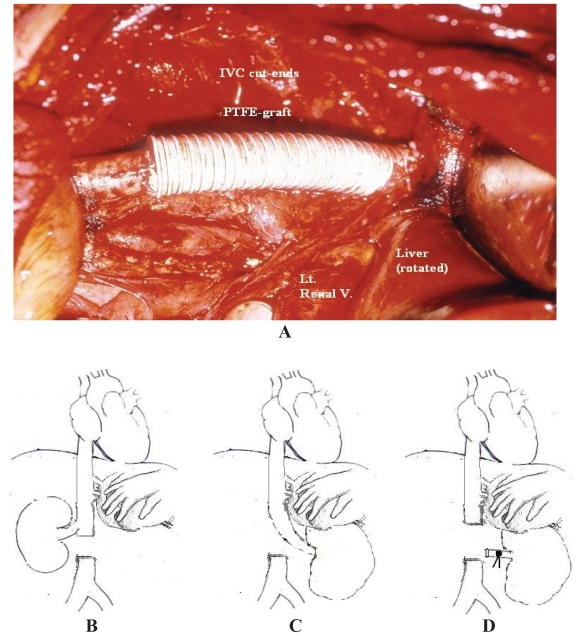
Options of resection of an invaded segment of subhepatic IVC. (**A**) Segment resection with reconstruction using PTFE graft. (B,C&D) Diagrammatic illustrations showing end-to-side right reno-caval anastomosis for left RCC (**B**), end-to-end left reno-caval anastomosis for right RCC (**C**) and IVC resection without reconstruction in case of the left renal vein due to abundant collaterals (**D**).

Subhepatic thrombi can be easily removed during radical nephrectomy through a cavotomy. However, if the tumor directly invades the caval wall or the thrombus is large, adherent and chronically obstructing the caval lumen, resection and primary reconstruction of infrahepatic IVC can be done with minimal postoperative morbidity using autologous (venous or pericardial) or synthetic (e.g. polytetrafluoroethylene (PTFE) grafts (Fig. 4-A).^6^ Prosthetic grafts are associated with a higher risk of thrombosis and infection.

Recent studies reported the possibility of excising a circumferential portion of infrahepatic IVC without reconstruction causing no morbidity or mortality directly related to surgery depending on an adequate collateral circulation.^1^,^18^ The major concern is the venous drainage of the remaining kidney. For the left renal vein, it can be ligated without reconstruction (Fig. 4-D) depending on its rich collateral circulation (adrenal, gonadal and lumbar veins) which can be accurately assessed intraoperatively by measuring its stump pressure (an adequate collateral circulation is associated with a stump pressure ≤50–60 cm. water (37–44 mmHg) but a higher pressure carries the risk of renal congestion or rupture). However, it is not the case for the right renal vein which lacks adequate collaterals and should be anastomosed end-to-end to the distal IVC segment (Fig. 4-B&C) or to the portal vein (if the distal IVC stump is short).^18^ Alternatively, a synthetic PTFE graft is used for anastomosis.^18^

### Group III

This group of caval thrombi poses a challenge to the surgeon due to its relative inaccessibility as the surgeon can not directly visualize the involved part of IVC.^6^ So, hepatic mobilization using liver transplantation (piggyback) technique is done to allow adequate exposure of the retrohepatic IVC.^1^ The hepatic ligaments are divided including the ligamentum teres, falciform ligament and the left triangular ligament. The visceral peritoneum is double-ligated and divided just below the inferior edge of the liver. Simultaneously the liver is gently rotated to the left, which helps to tent the right superior coronary ligament which is incised to rotate the liver to the midline, gradually exposing the retrohepatic IVC. Direct branches from the caudate lobe to the IVC should be carefully ligated so that the liver is attached to IVC only by the major hepatic veins and the retrohepatic IVC is completely visualized.

This maneuver will be sufficient for extracting group IIIa thrombi. For more cephalad levels of group III thrombi; the cephalic IVC clamp should be placed above the ostia of major hepatic veins which should be controlled by the Pringle’s maneuver i.e. application of a soft-bladed occlusive clamp across the porta hepatis.^1^ This will further impair the venous return and cause hepatic ischemia with consequent accumulation of toxic metabolites in the gut. However, it was reported that the tolerable normothermic continuous hepatic ischemic time is up to 15–30 minutes.^23^ Then tumor thrombi are extracted after creating a veno-venous bypass or CPB technique as previously described.

**Figure 5:**
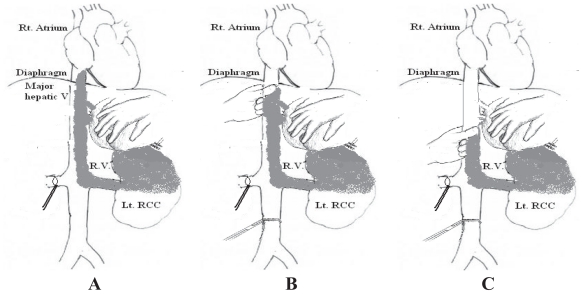
Diagrammatic illustration of steps of milking of IVC tumor thrombi below the level of major hepatic veins.

Ciancio et al. described a successful technique in removing these thrombi without intraoperative bypass maneuvers depending on milking of the tumor thrombus below the level of the major hepatic veins which is facilitated by IVC dissection from the posterior abdominal wall, thus allowing the surgeon fingers to wrap circumferentially around the IVC and avoiding letting loose a thrombus fragment (Fig. 5).^6^ This maneuver also helps in cases of Budd-Chiari syndrome as the resultant release of the congested liver is completely intravascular. However, if the thrombus is adherent to the caval wall or if IVC is left tethered to the abdominal wall, milking will exert traction on the IVC and may cause tearing of the caval wall which is a disastrous consequence. In such cases; it is safer to perform veno-venous bypass as IVC may require reconstruction.

### Group IV

In this group of tumor thrombi, the previous technique for group III thrombi is used added to CPB with or without DHCA to open the right atrium for extracting the tumor thrombus in a relatively bloodless field.^4^,^18^ Although these techniques are associated with significant perioperative morbidity and mortality due to the associated postoperative coagulopathy from full heparinization in CPB and neurological sequelae from DHCA, they are time tested and have proved useful but they should be used with caution.^6^ Modine et al. recently described a simplified minimally invasive technique for CPB in which the ascending aorta was canulated and the venous drainage was achieved using a proximal canula inserted in the superior vena cava and a distal canula inserted in the infra-renal IVC. Right atrial thrombus was extracted under normothermic CPB without cross clamping (just a temporary endoluminal occlusion catheter was introduced) or cardioplegic arrest.^24^

## Prognosis

The prognosis of RCC with IVC tumor thrombi is difficult to predict due to a wide variety in clinical behavior.^25^ Although involvement of the IVC in renal cancer is generally not a vascular invasion by the neoplastic process but mostly an intraluminal extension of the tumor mass, such intravascular growth implies a heightened biologic behavior of the tumor. Previous reports have shown that total resection of tumor affords the best chance of cure and long-term survival when no distant metastases are present.^4^ Due to the absence of effective alternative treatment, recent reports recommend surgical treatment even in the presence of distant metastases.^26^ With the advances in immunotherapy, survival of these patients may increase if aggressive surgery is combined with immunotherapy.^18^,^27^

The prognostic significance of IVC thrombi in RCC patients is still controversial. Furthermore, factors predicting the postoperative prognosis of RCC involving the IVC have not been well characterized.^28^ Numerous contemporary studies have demonstrated that tumor thrombus has a limited prognostic role in the absence of nodal and/or metastatic disease and that about 45%–70% of cases can be cured with surgical extirpation.^2^,^8^ Others showed that the prognosis of RCC with IVC thrombi is generally poor with 5-year survival rates about 25%–57%., despite surgical resection of RCC and tumor thrombus.^2^,^29^

Various centers have reported mortality rates of 2.7%–13% for IVC extension of RCC.^30^ The major cause are of deaths reported to be pulmonary embolism and myocardial infarction or due to complications related to the bypass procedures. However, with better perioperative management and standardization of the surgical techniques, the mortality rates can be decreased considerably.^4^

There is a controversy concerning the prognostic significance of the tumor thrombus level in the IVC.^31^ Some studies suggest that the risks of metastases and early death increase with a more cephalad extent of IVC thrombi.^32^ Skinner reported a 5-year survival rate of 35% after surgical treatment for patients with RCC and a subhepatic IVC thrombus, 18% for those with intrahepatic, and 0% for those with atrial tumor thrombi.^32^ Others found that in the absence of other adverse prognostic factors, the cephalad extension of IVC involvement is not prognostically important.^25^,^33^

It has been shown that the patients with RCC extending only into the IVC have significantly better survival rates than those with local spread of the tumor to the lymph nodes or perinephric tissue.^4^ However, whether the perinephric spread is the only factor to affect the prognosis adversely in patients with RCC with IVC extension or not is controversial.^34^ Libertino reported that perinephric spread, per se, does not affect the survival in patients with IVC thrombus.^34^ In contrast, others have reported that, the presence of perinephric spread in RCC is a strong poor prognostic parameter.^16^ In addition, there is also controversy concerning the prognostic significance of distant metastasis in patients with RCC and IVC thrombus. Although the presence of distant metastasis is generally regarded as a poor prognostic indicator in patients with RCC involving the IVC, there was no significant difference in survival between patients with and without distant metastasis in some studies.^35^

Patients with incomplete resections (excluding patients with metastatic disease) have a significantly worse prognosis, a finding that reflects the importance of surgical eradication of disease.^4^ Hatcher noted that the prognosis was determined by the ability to perform a complete resection of the tumor, not by the level of tumor thrombus.^36^,^37^

In conclusion, all patients with RCC and IVC tumor thrombi (even those with distant metastasis) should be considered for operation (as there is no other therapeutic modality). These tumors can be totally resected by an aggressive approach with an acceptable morbidity and mortality, and satisfactory long-term survival rates.^25^

## Comments and Future Perspectives

Removal of IVC tumor thrombi during radical or cytoreductive nephrectomy implicates an aggressive surgical technique that may increase the operative morbidity and mortality. In addition to the previously mentioned hazards of different techniques, the possibility of missed tumor thrombi during these maneuvers is obvious mostly because removal of the tumor thrombus under direct vision necessitates cavotomy which may be so long with more cephalad extension of the thrombus.

As an imaging modality, angioscopy provides a simple method for the careful evaluation and treatment of the lumen of native vessels and bypass grafts. With the recent advance in endovascular tools, angioscopically guided luminal intervention has become an increasingly useful approach to many vascular problems.^38^ The Angio-catheter can be used safely and successfully to remove thrombus from the native coronary artery of patients with myocardial infarction by rheolytic mechanisms.^39^ To date, no studies were evolved reporting the possibility of a therapeutic role for vascular endoscopy in IVC tumor thrombectomy.

We hereby represent a suggestion about angioscopic removal of such tumor thrombi under direct vision. Preoperatively, a vascular filter is percutaneously inserted just above the cephalic end of the tumor thrombus (up to the pulmonary artery in right atrial thrombi) to prevent thrombus embolization during the maneuver. After removal of the affected kidney, a short segment of the abdominal IVC is dissected free from the posterior abdominal wall (to facilitate complete cross clamping after collecting the tumor thrombi in the infra-hepatic IVC). The renal vein stump on the affected side is secured by a non-crushing vascular clip (to be used as an entry for the angioscopic port). An occlusive multi-channel balloon catheter is percutaneously introduced from the femoral vein up to the infra-renal IVC and a similar catheter is introduced from the internal jugular vein passing the SVC (superior vena cava) and the right atrium to be inflated in the upper end of the IVC. Both catheters are connected to a cell-salvage pump to which the distal catheter (venous limb) provides blood from both the infra- and supra-renal segments of IVC and the proximal catheter (inflow limb) returns a purified temperature-adjusted blood to the right atrium. By activation of this occlusive cell-salvaging veno-venous bypass system, the tumor thrombus bearing segment of IVC is completely occluded (except for a minimal flow from the major hepatic veins to allow better visualization of the caval wall and facilitates thrombectomy) without affecting the patient’s hemodynamics and without interrupting the hepatic vascular supply during the maneuver. The angioscope (larger than that used for coronary artery angioscopy) is introduced through a short port fixed in the stump of the renal vein or in a very small cavotomy (to allow re-introduction of the angioscope without back bleeding). The angioscope is advanced into the IVC to detect the level of the thrombus, its pathologic nature and the degree of caval wall involvement (whether it is just extending into the caval lumen, adherent or infiltrating the caval wall). If the thrombus is floating in the caval lumen or loosely adherent to the caval wall, it is gently sucked by a suction tube within the angioscope or dragged back to the abdominal IVC using a forceps or a multi-wired net basket. If the thrombus is infiltrating the caval wall, gentle trials to dissect it should be done then the thrombus is dragged back to the abdominal IVC. But, if these trials failed, exploration and resection of the vena cava with or without reconstruction is considered according to the level of caval involvement. The ostia of the major hepatic veins are inspected for any possible thrombus extension which can be removed concomitantly. The IVC lumen is then inspected for any residual thrombi (which can be collected and distally dragged). A suprarenal IVC clamp is now applied above the thrombi. Attempts are made to remove the thrombi through the port opening using a suction irrigation technique through the angio-catheter. But if the thrombus is large, it can be removed through a small cavotomy. The port is then removed, the bypass system is deactivated and renal vein stump (or the abdominal IVC cavotomy) is secured.

If the thrombus extends into the right atrium, the patient is shifted to a transient hypotensive state and the atrial thrombus is removed by the angio-scope by the same technique of caval thrombi (the angioscope can be guided by an esophageal echocardiography if visualization in the right atrium is difficult or hypotensive state is not preferred). Otherwise, it should be removed through an atriotomy using the simplified CPB technique previously described.^24^

This endoscopic removal of the tumor thrombi provides many advantages. At first, it allows direct visualization of the tumor thrombi and the caval lumen through out the technique which is not accessible in open surgical techniques. Second, it avoids the aggressive time-consuming dissection of IVC from the posterior abdominal wall and liver mobilization with their surgical consequences including defragmentation of tumor thrombi during the maneuver and the risk of postoperative renal or hepatic dysfunction. Third, it allows removal of thrombi in a relatively bloodless field. Fourth, it provides a reduction in incision length and soft-tissue dissection which can result in decreased patient morbidity and extended patient benefit also it can be done by a subcostal abdominal approach or after performing hand-assisted laparoscopic nephrectomy (only the abdominal IVC is needed to be accessed). Fifth, it avoids the complicated techniques and the surgical consequences of CPB and DHCA. In conclusion, management strategies for IVC thrombi in patients with RCC should be further studied to avoid major surgical trauma in such surgically compromised patients.
